# Time-of-Day and Age Impact on Memory in Elevated Plus-Maze Test in Rats

**DOI:** 10.3389/fnbeh.2018.00304

**Published:** 2018-12-06

**Authors:** Nicanor Morales-Delgado, Natalija Popović, Ernesto De la Cruz-Sánchez, María Caballero Bleda, Miroljub Popović

**Affiliations:** ^1^Department of Human Anatomy and Psychobiology, Faculty of Medicine, University of Murcia, Murcia, Spain; ^2^Institute of Biomedical Research of Murcia (IMIB), Virgen de la Arrixaca University Hospital, University of Murcia, Murcia, Spain; ^3^Department of Physical Activity and Sport, Faculty of Sport Science, University of Murcia, Murcia, Spain

**Keywords:** aging, circadian rhythm, elevated plus-maze, memory, rat

## Abstract

The purpose of the present study was to establish the effect of daytime and aging on memory in rats in the Elevated Plus-Maze (EPM) test. Young (2-months) and aged (18-months) male Wistar rats were exposed to the EPM test, at the beginning, mid-time or at the end of the light period. On the acquisition trial, the animals were placed individually at the end of one of the open arms of the EPM and the latency to enter in the enclosed arms was registered (cut-off time 60 s). The test was repeated 24 h later on. A longer latency period to reach the enclosed arm indicated poor retention compared to significantly shorter latencies. There were no significant differences between groups on the acquisition trial. In all tested periods, the latency time on the 24 h retention trial was significantly shorter in the young rats compared to the old ones. Furthermore, in the early and mid-time period of the light period, the young rats showed significantly decreased transfer latency (TL) time on the 24 h retention trial in comparison with the acquisition trial. In the aged rats, the TL time on the 24 h retention trial was significantly longer at the end of the light period, in comparison to the two other testing periods. In conclusion, aging significantly affects memory and the more critical period for memory process in both young and old animals, particularly at the end of the light period of the circadian cycle.

## Introduction

The extant literature indicates that spatial navigation and spatial memory, ones of the crucial abilities for everyday living and survive, decline with aging, in rodents, non-human primates and humans (Foster et al., 2012; Klencklen et al., 2012; Colombo et al., 2017; Lester et al., 2017). Although the impact of time-of-day on cognitive function has been matter of interest for many years (Gerstner and Yin, 2010), there are scarce data on the relationship between spatial memory, aging and time-of-day in rodents. Several studies indicate that young rodents performs better during the active phase than during the rest period, in different spatial memory tasks, such as Morris water maze (Gritton et al., 2012; Martin-Fairey and Nuñez, 2014), 8-arm radial maze (Hauber and Bareiß, 2001), six point alley-T-maze (Hoffmann and Balschun, 1992), context-dependent fear conditioning (Valentinuzzi et al., 2001) and novel location recognition tasks (Takahashi et al., 2013). However, the time-of-day impact has not been demonstrated when rats were tested at the beginning and at the end of the light period, in the context-dependent fear conditioning task and Morris water maze (McDonald et al., 2002). In contrast, Winocur and Hasher (2004) found that young rats tested within 1 h after the beginning of the dark period, performed worsen a non-matching-to-sample variant of the water maze, than a group tested within 1 h before the end of dark period. However, the reverse situation was demonstrated in old rats.

The Elevated Plus-Maze (EPM) has been initially used as a test of anxiety in rats (Pellow et al., 1985) and mice (Lister, 1987) and shortly after as a test to evaluate memory in mice and rats (Itoh et al., 1990; Sharma and Kulkarni, 1992). Using this test, an age-related memory decline was found in rats (Haider et al., 2014; Nade et al., 2015) and mice (Reddy and Kulkarni, 1998; Carrié et al., 1999; Raghavendra and Kulkarni, 2001; Parle and Dhingra, 2003; Patil et al., 2003; Singh et al., 2003; Joshi and Parle, 2006a,b; Bansal and Parle, 2011). In both species, the animals were tested during the rest (light) phase of the circadian rhythm without tendency to evaluate time-of-day effect. The purpose of the present study was to evaluate the effects of aging and daytime of the light period, on memory in the EPM test.

## Materials and Methods

### Animals

The animal maintenance (three rats per cage; 22 ± 1°C room temperature; 30% humidity; food and water available *ad libitum*, lights on from 09:00 h to 21:00 h) and experimentation on 36 young (2-months, body weight: 242.3 ± 11.0 g) and 48 old (18-months, body weight: 864.9 ± 128.1 g) male Wistar rats were proceeded in accordance with the European Communities Council Directive of November 24, 1986 (86/609/EEC) and the guidelines issued by the Spanish Ministry of Agriculture, Fishing and Feeding (Royal Decree 1201/2005 of October 21, 2005) and were approved by the Institutional Animal Ethics Committee. All rats were individually handled for 2 days × 2 min, before the beginning of the training day. The EPM test was performed during the light period: in the morning (10:00–11:30 h; defined as Zeitgeber time (ZT) ZT1–2.5), early afternoon (14:00–15:30 h, defined as ZT5–6.5) and late afternoon (19:00–20:30 h, defined as ZT10–11.5). All efforts were made to minimize the number of animals used and their suffering.

### Elevated Plus-Maze (EPM)

EPM apparatus was made of black plywood and consisted of both two opposed open and enclosed arms (50 cm length × 10 cm width × 40 cm high for closed arms), and an open central square (10 cm length × 10 cm width). The maze was elevated 80 cm above the floor. On the acquisition trial, the rats were placed individually at the end of the open arm, facing it away from the central platform. The time that the animal took to move from the open arm to either one of the enclosed arms was registered by two experimenters. The entry into the enclosed arm was recorded when an animal’s all four paws passed the line dividing the central square from the open arms. The same procedure was repeated on the 24 h retention trial. Each animal that on the acquisition trial failed to enter the enclosed arm within 60 s or that fell off the maze (either during acquisition or retention trial) was excluded from the experiment. The criterium was achieved by 32 young animals (10, 10 and 12 at the ZT1–2.5, ZT5–6.5 and ZT10–11.5, respectively) and by 24 old rats (7, 9 and 8 at the ZT1–2.5, ZT5–6.5 and ZT10–11.5, respectively). To become acquainted with the EPM, the rats were allowed to explore the apparatus for 20 s after reaching the enclosed arm and then returned to its home cage. A longer transfer latency (TL) period to reach the enclosed arm on the second trial indicated poor retention compared to significantly shorter latencies. The apparatus was wiped clean with 70% ethanol before testing each animal.

### Statistical Analysis

Descriptive data are presented as mean, median and percentiles (P10, P25, P75 and P90). The statistical analysis was performed using SPSS 24.0 software (IBM Corp., Armonk, NY, USA). A General Linear Model repeated measures analysis was run to evaluate the effects of trails, age and time-of-day on TL, examining also two-way and simple main effects. In order to normalized data distribution natural logarithmic transformation was applied before the statistical analysis. Statistical significance was accepted at a Bonferroni-adjusted alpha level of 0.025 or 0.05, depending on the number of the comparisons made. TL comparison between the acquisition and the 24 h retention trials, in each time of the day, for each group (young and old animals), was done using a paired sample *t*-test.

## Results

The General Linear Model repeated measures analysis showed significant within-subjects effect of trial on TL (*F*_(1,50)_ = 17.603, *p* = 0.0001) and trial and age (*F*_(2,50)_ = 7.358, *p* = 0.009), but not an interaction of trial, age and time of the day (*F*_(2,50)_ = 0.151, *p* = 0.861) and trial and time-of-day (*F*_(2,50)_ = 1.372, *p* = 0.263). The same analysis showed significant between-subjects effect on TL and age (*F*_(1,50)_ = 45.697, *p* = 0.0001), TL and time-of-day (*F*_(2,50)_ = 4.471, *p* = 0.016) but not on TL, age and time-of-day (*F*_(2,50)_ = 0.860, *p* = 0.429).

**Figure 1:**
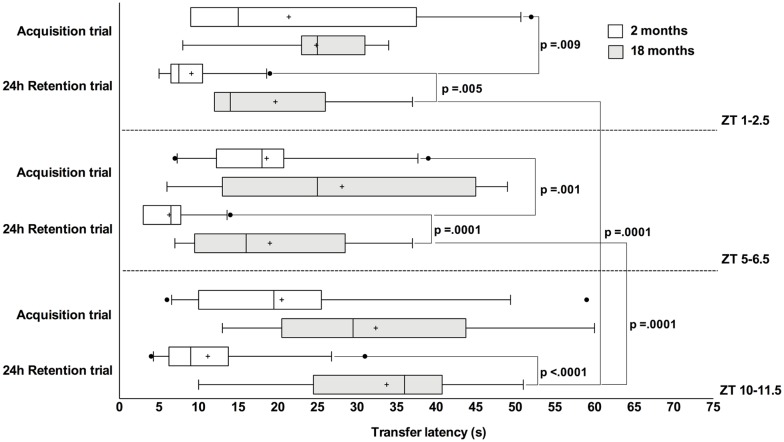
A long transfer latency (TL) period indicated poor retention compared to shorter latencies. Box-and-whisker plot showing median (vertical line inside box), mean (plus symbol), 25 and 75 percentiles (edge of box), 10 and 90 percentiles (whiskers) and extreme individual data points (out of box circle); significant pairwise comparisons are noted.

At the acquisition trial there was not statistically significant simple main *effect of the time-of-day on TL* in both young and old rats (*F*_(2,50)_ = 0.005, *p* = 0.995, *F*_(2,50)_ = 0.452, *p* = 0.639, respectively) and *effect of age in each time of the day on TL* as follows: ZT1–2.5 (*F*_(1,50)_ = 0.903, *p* = 0.347); ZT5–6.5 (*F*_(1,50)_ = 1.067, *p* = 0.306) and ZT10–11.5 (*F*_(1,50)_ = 3.855, *p* = 0.055). However, at the 24 h retention trial there was a statistically significant simple main *effect of the time-of-day on TL* in old rats (*F*_(2,50)_ = 3.355, *p* = 0.043), but not in the young ones (*F*_(2,50)_ = 3.116, *p* = 0.053). In the old animals, the TL at the end of rest period was significantly higher in comparison to the ZT1–2.5 (*p* = 0.0001) and ZT5–6.5 session (*p* = 0.0001; Figure 1). The statistically significant simple main *effect of age in each time of the day on TL* was found as follows: ZT1–2.5 (*F*_(1,50)_ = 8.572, *p* = 0.005), ZT5–6.5 (*F*_(1,50)_ = 19.827, *p* = 0.0001) and ZT10–11.5 (*F*_(1,50)_ = 23.201, *p* = 0.0001) indicating that TL at the 24 h retention trial was significantly higher in old rats vs. young ones in all tested periods (Figure 1).

The paired sample *t*-test reveals statistical differences between acquisition and 24 h retention trials for young rats tested at the ZT1–2.5 (*t*_(9)_ = 3.342, *p* = 0.009) and at the ZT5–6.5 period (*t*_(9)_ = 4.833, *p* = 0.001), but not at the ZT10–11.5 period (*t*_(11)_ = 1.923, *p* = 0.081). There were no statistical differences between trials in old rats tested in the ZT1–2.5 (*t*_(6)_ = 1.100, *p* = 0.314), ZT5–6.5 (*t*_(8)_= 0.879, *p* = 0.405) and ZT10–11.5 (*t*_(7)_ = 0.177, *p* = 0.864).

## Discussion

The results from the present study reveal that during the acquisition trial there are no significant differences in TL between aged and young rats. Moreover, neither in young or aged animals there were differences between three tested periods (ZT1–2.5; ZT5–6.5 and ZT10–11.5), suggesting altogether that there were no significant effects of the age and the time-of-day on motor or visual abilities nor motivation to escape from the open arm in the EPM. Similarly, Raghavendra and Kulkarni (2001); Jain et al. (2002) and Patil et al. (2003) did not found significant differences in the performance of the acquisition trial between 14 months and 16 months and 3 months aged mice. However, some other studies, demonstrated significantly higher TL on acquisition trial of 14–16 months aged mice (Reddy and Kulkarni, 1998; Singh et al., 2003) and 10–12 months aged Sprague-Dawley rats (Sharma and Kulkarni, 1992) than in the corresponding 2–3 months young animals.

Independently of the time of the day when the EPM test was performed: between 08:00 h and 15:00 h (Singh et al., 2003; Bansal and Parle, 2011), 10:00 and 13:00 h (Raghavendra and Kulkarni, 2001) or 09:00–18:00 h (Reddy and Kulkarni, 1998; Carrié et al., 1999; Jain et al., 2002), the TL from the open to the enclosed arm on 24 h retention trial was higher in 14–18 months aged mice than in the 2–3 mice young ones. Moreover, the TL between trials significantly decreased in young but not in the old animals (Raghavendra and Kulkarni, 2001; Jain et al., 2002; Singh et al., 2003). The longitudinal studies performed in young (3 months) and aged (16 months) Swiss mice showed faster forgetting in aged than in young animals (Patil et al., 2003).

In the EPM test performed in the light period, aged Wistar rats (22–24 months) exhibited significantly longer TL on the 24 h retention trial compared to the young rats (4–5 months; Haider et al., 2014). In the present study, independently of the ZT period, the TL on the 24 h retention trial was significantly shorter in the young Wistar rats, compared to the old ones suggesting deficit in spatial memory in the aged animals. Curiously, in opposite to our and all previously mentioned findings, Sharma and Kulkarni (1992) found that during the light period (09:00–12:00 h) the TL significantly decrease on the 24 h retention trial in 10–12 months but not in the 2–3 months aged Sprague-Dawley rats.

In contrast to the data of McDonald et al. (2002) that showed no differences between rats (strain and age not specified) tested at the beginning (ZT2) and at the end of the light period (ZT11) in the Morris water maze task, the present study indicates that in both groups, young and aged Wistar rats, the end of the light period maybe considered as the more vulnerable period for memory formation in the EPM test. Namely, in young rats, only during the beginning and mid-time period of the light period occurred significant decrease in the TL between trials, while in the aged rats, the TL time on the 24 h retention trial was significantly longer at the end of the light period in comparison to the two other testing periods. Similarly, in humans, the worsen period to perform cognitive tasks is at the end of the resting period (early morning hours: from 3 am until 7 am; Evans et al., 2017). Since sleeping helps the consolidation of spatial memory (Vorster and Born, 2015), the possibilities of memory consolidation disruption could be higher at the end of the resting period than at the beginning and mid-time of the same period.

## Conclusion

Aging significantly affects memory in the EPM test and it seems that during the light phase of the circadian cycle, its ending period maybe the more critical period for memory process in both young and old rats.

## Data Availability

The datasets generated for this study are available on request to the corresponding author.

## Author Contributions

NM-D, NP, EC-S, MCB and MP contributed to the design of the study, wrote the protocol, managed the literature searches, contributed to drafting the work and have approved the final manuscript. NM-D, NP and MP performed the experiments. EC-S and MP undertook the statistical analysis.

## Conflict of Interest Statement

The authors declare that the research was conducted in the absence of any commercial or financial relationships that could be construed as a potential conflict of interest.
